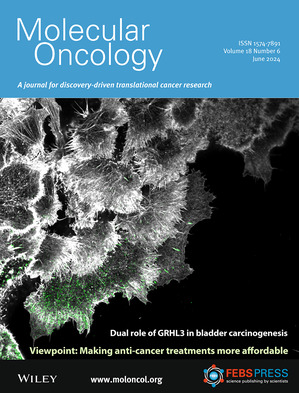# Issue Information

**DOI:** 10.1002/1878-0261.13460

**Published:** 2024-06-08

## Abstract

Although discoveries in biomedical research power the development of personalised and more effective anti‐cancer treatments, access to these treatments may become limited especially for less privileged communities. Thus, there is an urgent need to re‐organize and re‐assess drug development and cost‐effectiveness. Read the full *EACR Viewpoint* article by Anton JM Berns on how academia and society should join forces to make anti‐cancer treatments more affordable in pp. 1351–1354.

**On the cover:** Confocal images of the actin cytoskeleton (white) and focal adhesions (green) of SCaBER bladder cancer cells upon migration. Read the full article by Franziska C. Lammert *et al*. in pp. 1397–1416.

**Illustration credits:** Florian Friedland and Dr. Erik Noetzel‐Reiss, Institute of Biological Information Processing 2, Mechanobiology, Forschungszentrum Jülich GmbH.